# Transcriptional regulation of dosage compensation in *Carica papaya*

**DOI:** 10.1038/s41598-021-85480-3

**Published:** 2021-03-12

**Authors:** Juan Liu, Jennifer Han, Anupma Sharma, Ching Man Wai, Ray Ming, Qingyi Yu

**Affiliations:** 1 Center for Genomics and Biotechnology, Fujian Provincial Key Laboratory of Haixia Applied Plant Systems Biology, Haixia Institute of Science and Technology, Fujian Agriculture and Forestry University, Fuzhou, Fujian Province China; 2 Texas A&M AgriLife Research Center at Dallas, Texas A&M University System, Dallas, TX USA; 3 Department of Plant Biology, University of Illinois at Urbana-Champaign, Urbana, IL USA; 4 Department of Plant Pathology and Microbiology, Texas A&M University, College Station, TX USA

**Keywords:** Plant evolution, Molecular evolution, Genomics, Evolution, Plant sciences

## Abstract

Sex chromosome evolution results in the disparity in gene content between heterogametic sex chromosomes and creates the need for dosage compensation to counteract the effects of gene dose imbalance of sex chromosomes in males and females. It is not known at which stage of sex chromosome evolution dosage compensation would evolve. We used global gene expression profiling in male and female papayas to assess gene expression patterns of sex-linked genes on the papaya sex chromosomes. By analyzing expression ratios of sex-linked genes to autosomal genes and sex-linked genes in males relative to females, our results showed that dosage compensation was regulated on a gene-by-gene level rather than whole sex-linked region in papaya. Seven genes on the papaya X chromosome exhibited dosage compensation. We further compared gene expression ratios in the two evolutionary strata. Y alleles in the older evolutionary stratum showed reduced expression compared to X alleles, while Y alleles in the younger evolutionary stratum showed elevated expression compared to X alleles. Reduced expression of Y alleles in the older evolutionary stratum might be caused by accumulation of deleterious mutations in regulatory regions or transposable element-mediated methylation spreading. Most X-hemizygous genes exhibited either no or very low expression, suggesting that gene silencing might play a role in maintaining transcriptional balance between females and males.

## Introduction

Dioecy accelerates adaptive evolution through promoting outcrossing and increasing genetic variation^1^. Plant sex chromosome needs two mutations, both male sterility and female sterility genes, to evolve from hermaphroditic ancestral status for dioecy evolution^2^. In XY system, female is homogametic (XX) and male is heterogametic (XY), while in ZW system, female is heterogametic (ZW) and male is homogametic (ZZ)^3^. Genes in the non-recombining region of Y or W chromosome undergo genetic degeneration, which causes a difference in gene dose between homogametic and heterogametic sexes^4,5^. Loss of a few genes might be tolerated for heterogametic sex, but massive gene loss is usually deleterious. Ohno proposed that dosage compensation would evolve to compensate the aneuploidy caused by genetic degeneration of Y or W in heterogametic sex^6^. Dosage compensation functions in two aspects. It balances the expression between sex-linked genes and autosomal genes, which can be critical to maintain fitness when sex-linked genes are dosage sensitive. It also equalizes the expression of sex-linked genes between males and females. Dosage compensation can occur globally or locally^7–9^. Global dosage compensation has evolved in several species, among them *Drosophila*, placental mammals and *Caenorhabditis elegans* are model species in which mechanism of dosage compensation is well studied and understood^4^. To date, most of the knowledge on dosage compensation comes from these model species^9–11^. However, global dosage compensation is relatively rare, and largely confined to species with highly degenerate Y chromosomes.

Plants often have younger sex chromosomes than animals, providing opportunities to study dosage compensation in early stages of sex chromosome evolution^3,12–15^. Sex chromosomes of *Silene latifolia* arose approximately 11 MYA^16^. In *S. latifolia*, significant differences in expression of X hemizygous genes (X-linked genes without Y alleles) were observed between male and female plants, but no expression difference was observed in X-linked genes with preserved Y alleles^12,13,17^. Hemizygous X-linked genes can be divided into two groups based on their expression ratios between male and female (mX/fXX), one with the ratio approximately at 1 and the other one with the ratio at 0.5, indicating that only a subset of X-linked genes displayed dosage compensation^14^. For the X-linked genes with expressed Y alleles, the expression ratio increased as the Y alleles decreased expression. Similar distribution of mX/fXX was observed among various levels of X/Y divergence, suggesting that dosage compensation and X/Y divergence evolved independently^14,18,19^. Partial dosage compensation was also observed in *Rumex hastatulus* that has cytologically distinguishable heteromorphic sex chromosomes^15^. The expression ratio of Y alleles relative to X alleles in male was significantly lower in the old evolutionary stratum than the one in the young evolutionary stratum, whereas the total expression of Y and X alleles in these two strata showed no significant reduction relative to the total expression of two alleles in female, suggesting that dosage compensation probably coevolved with X/Y divergence during sex chromosome evolution in *R. hastatulus*^15^. These studies in plants revealed that dosage compensation operates only on some genes instead of the entire non-recombining region. Genes in non-recombining regions degenerate gradually instead of becoming nonfunctionalized instantly during the evolution of sex chromosomes. Therefore, a gene-by-gene analysis is necessary to fully understand the evolutionary process of dosage compensation, especially for the young sex chromosomes in plant species. Sex chromosomes of papaya evolved 7 MYA, younger than the ones in *R. hastatulus* and *S. latifolia*^15,20^. With the complete genome sequence and annotation of non-recombining regions of sex chromosomes available, papaya offers an unprecedented opportunity to study effects of dosage compensation at early stages of sex chromosome evolution^20,21^.

Papaya (*Carica papaya*) belongs to the family Caricaceae that contains six genera and 35 species. Among the 35 species, *Vasconcella monoica* is the only monoecious species that does not have sex chromosomes, while all the other species are either trioecious or dioecious^20,22^. Papaya is trioecious with three sex types (female, male, and hermaphrodite) controlled by a XY system. The two Y chromosomes controlling the development of hermaphrodites (Y^h^) and males (Y) are slightly different^21,23,24^. The non-recombining regions of papaya sex chromosomes have been fully sequenced using a BAC (Bacterial artificial chromosome)-by-BAC strategy^21,23,24^. Two evolutionary strata, corresponding to two inversions on the Y chromosome, were identified in the papaya non-recombining region. The non-recombining region of the papaya Y chromosome is 8.1 Mb whereas its counterpart of the X chromosome is 3.5 Mb^21^. All the genes in the non-recombining regions have been annotated, making it possible to evaluate dosage compensation on a gene-by-gene level and to study the relationship between dosage compensation and evolutionary strata.

## Results

### Expression of genes in non-recombining regions of papaya sex chromosomes

In the non-recombining regions of papaya X and Y chromosomes, 50 X/Y paired genes, 34 X-specific genes, and 22 Y-specific genes were annotated^21^. A straightforward test for dosage compensation is to compare the average expression of X-specific genes to the average expression of autosomal genes in males (mX/mAA). In general, dosage compensation is considered to be evolved if the ratio of mX/mAA is close to 1. However, among the 34 X-linked hemizygous genes without Y alleles (X-specific genes), only two of them were expressed in male flower tissues and one expressed in male leaf tissues, making it impossible to test dosage compensation in papaya using this method. We therefore chose X/Y paired genes (including pseudogenes) in the non-recombining region to test dosage compensation in papaya.

### Expression ratios of X-linked genes to autosomal genes in male papaya tissues

**Figure 1:**
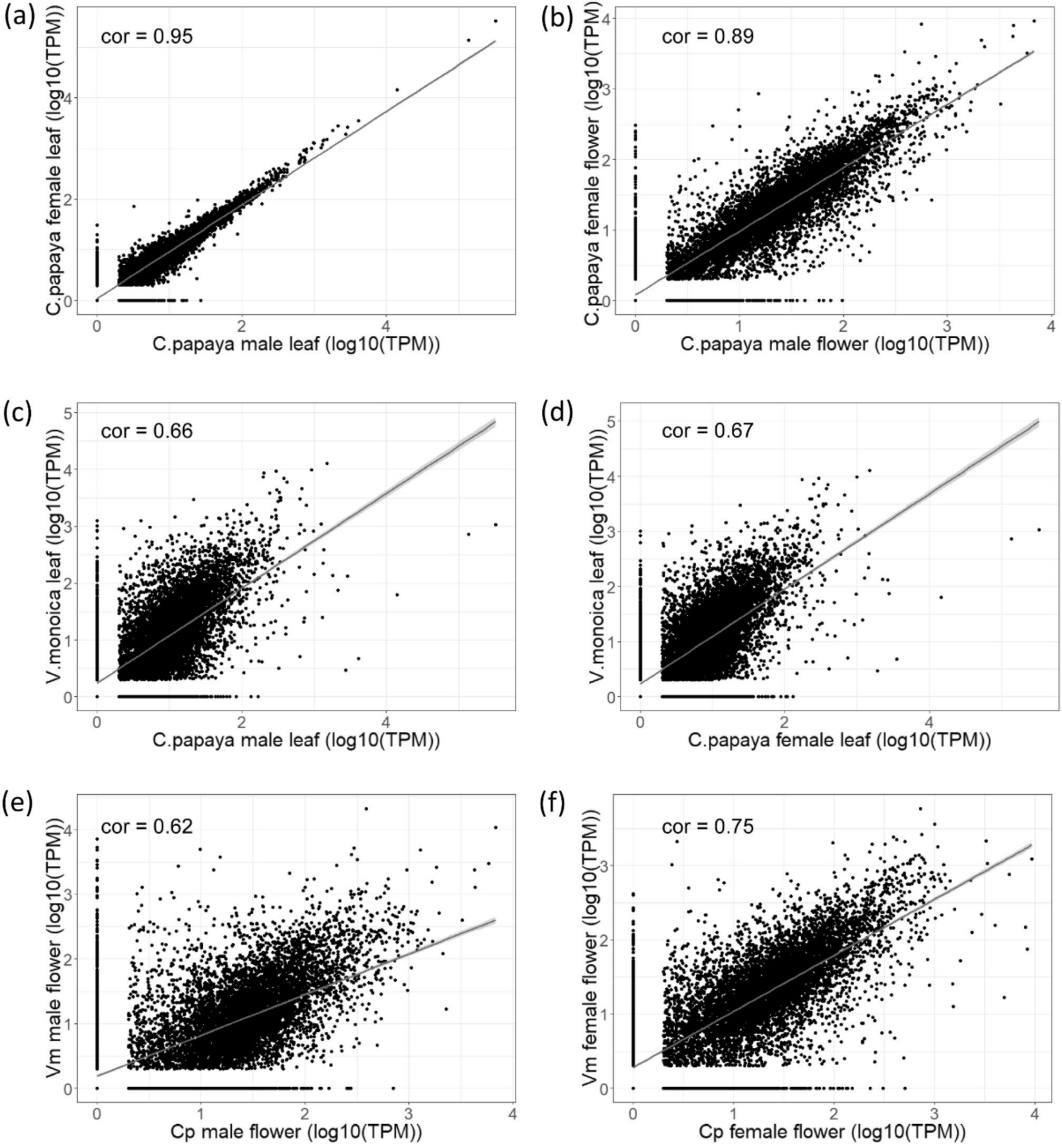
Plot of gene expression (log_10_ (TPM)) between male and female papaya leaves (**a**), between male and female papaya flowers (**b**), between *V. monoica* leaf and papaya male and female leaves (**c**,**d**), and between *V. monoica* and papaya male and female flowers (**e**,**f**). The best fit line was draw in blue. Correlated rate was estimated by Pearson’s correlation.

We used RNA-Seq data of papaya male and female leaf and flower tissues to study whether dosage compensation has evolved in papaya. The global gene expression patterns were highly correlated between male and female in both leaf (Fig. 1a) and flower (Fig. 1b) tissues. However, expression levels of sex-linked genes were significantly lower than that of autosome genes (Wilcoxon rank sum test, *p* < 0.05) in both leaf and flower tissues (Supplementary Fig. S1). We then chose *V. monoica*, a close relative species of papaya without sex chromosomes, to calibrate the expression of genes on sex chromosomes and autosomes.

First, we compared the global gene expression between papaya and *V. monoica*. Overall, the gene expression in both male and female papaya leaves was correlated with their orthologs in *V. monoica* (Spearman’s rank correlation, male rho = 0.66, N = 10,010 genes, *p* < 0.001; female rho = 0.67, N = 10,010 genes, *p* < 0.001) (Fig. 1c,d). The result demonstrated that most of the genes in papaya and *V. monoica* had conserved expression patterns. Similar correlation was also observed in both male and female flowers between papaya and *V. monoica* (Fig. 1e,f).

After calibration of gene expression using *V. monoica*, we compared median expressions of genes in sex-specific regions and genes on autosomes. No significant difference of median expression was detected between sex-specific region and autosome genes in either male leaf (Wilcoxon rank sum test, W = 253,910, *p* value = 0.352) or male flower (Wilcoxon rank sum test, W = 291,850, *p* value = 0.3981) tissues (Table 1). The calibrated median sex-specific region/Autosome expression ratios were 1.064 and 1.125 in male leaf and male flower tissues, and 1.141 and 1.000 in female leaf and female flower tissues, respectively (Table 1). However, the calibrated mean sex-specific region/Autosome expression ratios were 0.502 and 0.842 in male leaf and male flower tissues, and 0.661 and 0.985 in female leaf and female flower tissues, respectively. Our result showed dosage imbalance between sex-specific region and autosome genes, and between female and male papayas. In general, genes in sex-specific region showed reduced expression in male tissues compared to their counterpart female tissues. However, the reduction was less than 50%, 24% in leaf tissue and 14.5% in flower tissue, indicating that partial compensation or compensation in some genes may exist. Different degrees of reduction in flower and leaf tissues may suggest that dosage compensation in papaya varies among tissues.

**Table 1:** The median and mean expression ratios of genes in sex-specific region to autosome genes in leaf and flower tissues of papaya male and female plants.

Sample	Sex-specific		Autosome		Sex-specific/autosome	
	Median	Mean	Median	Mean	Median	Mean
Male leaf	1.000	0.988	0.940	1.970	1.064	0.502
Female leaf	1.000	1.230	0.877	1.861	1.141	0.661
Male flower	2.224	3.470	1.976	4.121	1.125	0.842
Female flower	1.000	2.246	1.000	2.280	1.000	0.985

### Expression ratio of sex-linked genes in male relative to female

**Figure 2:**
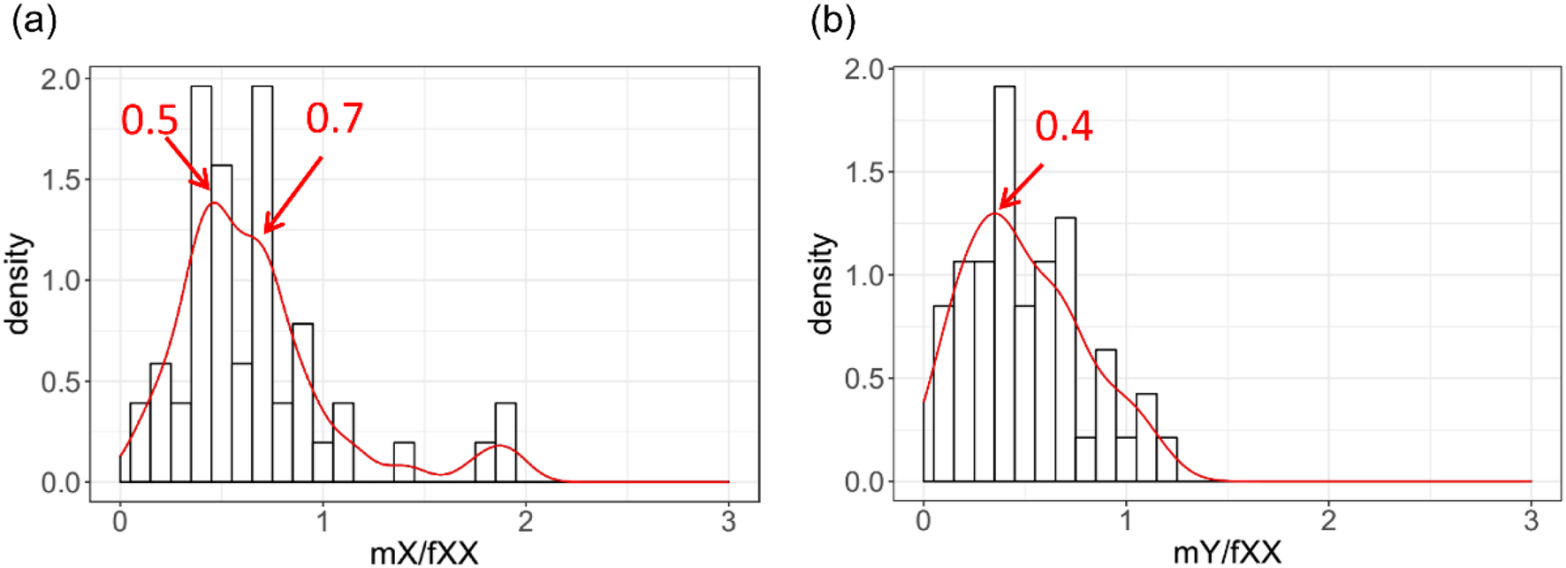
The distribution of relative transcript abundance of X-alleles (**a**) and Y-alleles (**b**) in male relative to their corresponding X alleles in female. SNPs were called by GATK Haplotypecaller and read counts were normalized by number of SNPs and the library size of each sample. Outliners are not shown. Bin size in histogram is 0.1. Red curve shows the kernel-smoothed density estimation. Peaks are highlighted with red arrows.

One of the major aims of dosage compensation is to mitigate the effects of imbalanced gene dose in males and females. If no dosage compensation evolved, X and Y alleles are expected to show equalized expression in male and their average expression in male should be half of the expression of the two X alleles in female (mX = mY = 0.5fXX). If partial dosage compensation exists, mX > mY and mX > 0.5fXX. The average expression ratios of genes in sex-specific region between male and female (mXY/fXX) were initially calculated at 1.00 and 1.16 in leaf and flower tissues, respectively. Since X and Y alleles were highly identical, misalignment could happen during mapping reads to the reference genome sequence for male samples. For this reason, we recalculated the expression ratios using single-nucleotide polymorphisms (SNPs) to distinguish X and Y alleles. We also normalized read counts by total reads of each library, making it comparable across male and female libraries. The median expression ratios of mY/mX in leaf and flower tissues were 0.81 and 0.92, respectively, indicating decreased expression of Y alleles compared to their corresponding X alleles. A double-peaked distribution was observed for expression ratios of X alleles in male relative to female (Fig. 2a). A high peak was observed at 0.5, indicating a large number of X alleles are dosage uncompensated. The secondary peak was observed at 0.7 with a long tail reaching above 1, indicating partial dosage compensation has evolved for a subset of genes. In contrast, the expression ratio of Y alleles in male relative to their corresponding X alleles in female was centered at 0.4, lower than 0.5, indicating reduced gene expression of Y alleles (Fig. 2b).

**Figure 3:**
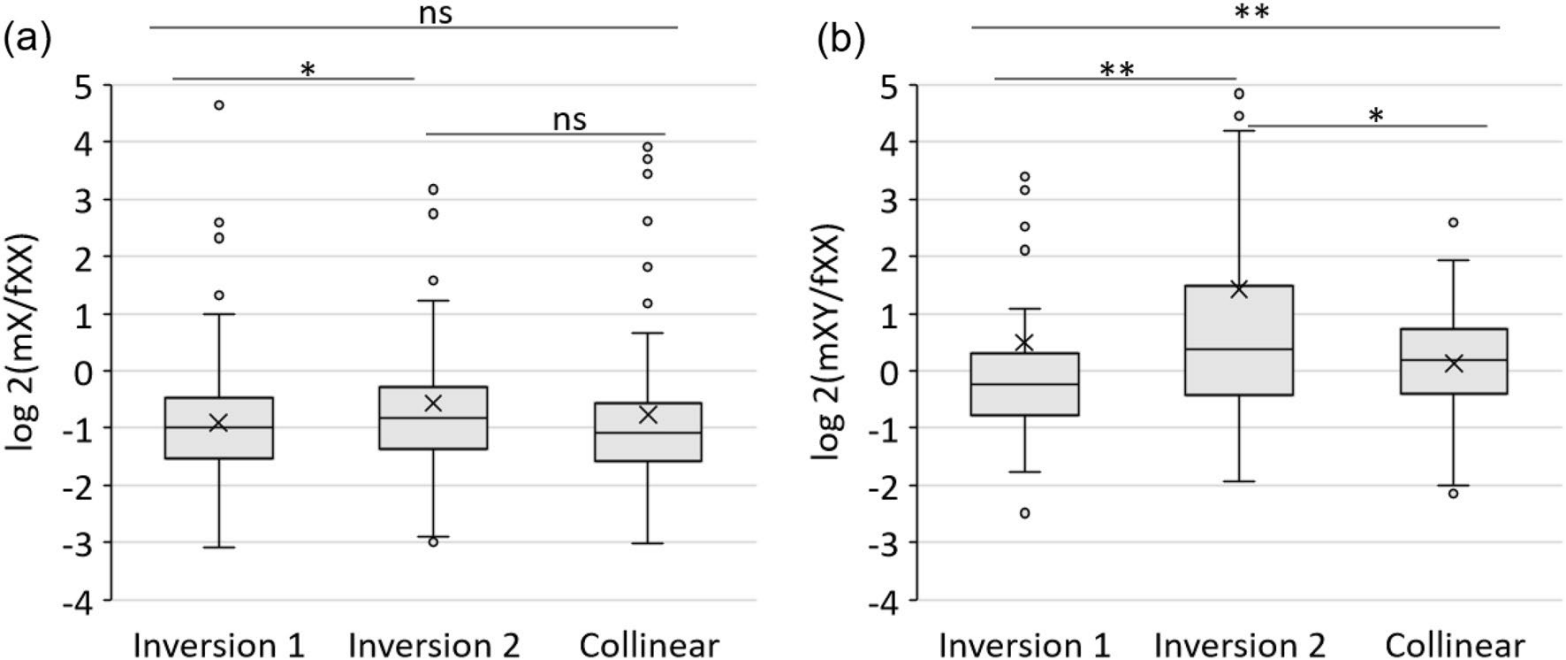
Boxplot of the log_2_ transformed expression ratios of mX/fXX (**a**) and mXY/fXX (**b**) of genes on two different evolutionary strata in papaya male samples. Mann–Whitney’ test was used for statistics analysis. **p < 0.01; *p < 0.05, ns: no significant. The line halfway the plot marks the median for each box. The X in the box represents the mean for each box.

We further analyzed expression ratios of genes in the two evolutionary strata (corresponding to inversion 1 and inversion 2) and the collinear region. In the collinear region, the median log_2_ transformed expression ratio of X alleles in male relative to female (mX/fXX) was around − 1 and the log_2_ (mXY/fXX) was around 0, indicating that the expression of X alleles in male was about half of the expression of two X alleles in female and X and Y alleles shared similar levels of expression (Fig. 3a,b). In the inversion 1, the log_2_ (mX/fXX) was around − 1 and the log_2_ (mXY/fXX) was below 0, indicating that the expression of X allele in male was about half of the expression of two X alleles in female but Y alleles had reduced expression compared to their corresponding X alleles (Fig. 3a,b). In the inversion 2, the log_2_ (mX/fXX) was slightly above − 1, but the log_2_ (mXY/fXX) was above 0, significantly higher than the ones in the collinear region and the inversion 1, suggesting elevated expression of Y alleles relative to their corresponding X alleles in the inversion 2 (Fig. 3a,b).

Although dosage compensation of whole sex-linked region was not observed in papaya, local compensation may exist. We analyzed the mX/fXX ratio of all expressed genes one by one in eight different tissues, including three leaf and five flower tissues (Supplementary Fig. S2). One gene in the inversion 1, *PYhCpXYh7* (its Y-allele is a pseudogene) showed dosage regulation, with a median expression ratio of mX/fXX at 0.986. Four genes in the inversion 2, *CpXYh18*, *CpXYh23*, *CpXYh29* and *CpXYh32*, and two genes in the collinear region, *CpXYh35* and *CpXYh38*, had a mX/fXX ratio close to 1 or higher than 0.75, suggesting that these genes were dosage compensated or partially dosage compensated. We also calculated the expression ratio of total X and Y alleles in male relative to the two X alleles in female. The log_2_ (mXY/fXX) of most of the genes mentioned above were higher than zero (Supplementary Fig. S3).

### Gradual reduction of Y allele expression with sex chromosome evolution

**Figure 4:**
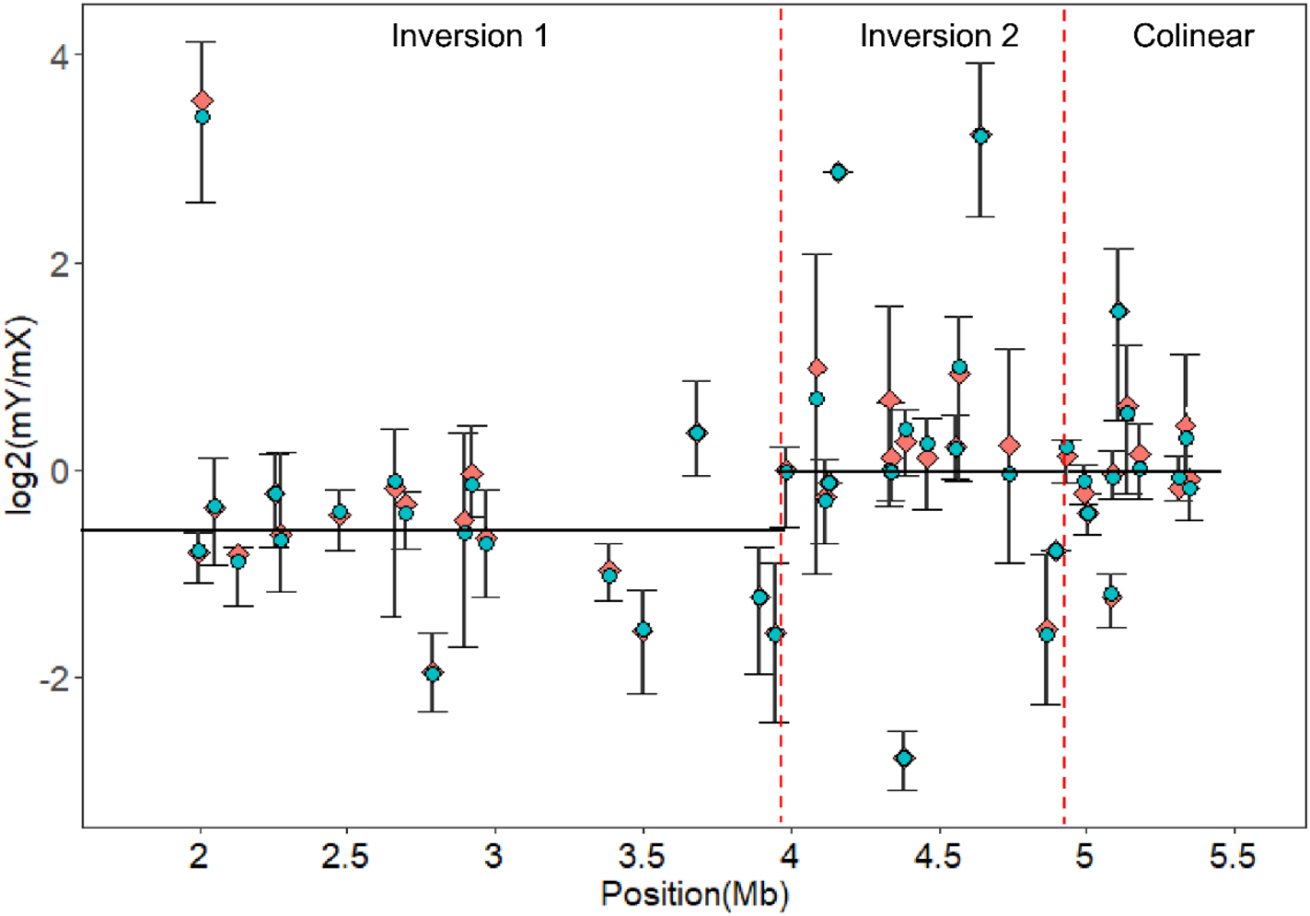
The distribution of log_2_ transformed expression ratio of Y alleles relative to X alleles in male samples (including three leaf replicates and five flower replicates) across the non-recombining region. Forty-five pairs of expressed genes were used in the analysis, including 18 genes in inversion 1, 16 genes in inversion 2, and 11 genes in the collinear region. The X-axis represents the position of genes in the non-recombining region of the X chromosome. Cyan and orange dots represent the log_2_ value of average and median ratios between Y and X alleles, respectively.

We calculated expression ratios of Y alleles relative to their corresponding X alleles in male leaf and flower tissues. In the inversion 1, an older evolutionary stratum, Y alleles showed a relatively lower level of expression than X alleles (Fig. 4, Supplementary Fig. S4). In the younger evolutionary stratum inversion 2 and the collinear region, X and Y alleles showed a similar level of expression. In general, the expression ratio of Y alleles relative to X alleles was related with evolutionary strata of the non-recombining region in papaya with few exceptions (Fig. 4). *CpXYh2* in inversion 1 and *CpXYh29* in inversion 2 showed Y-biased expression. Consistent with the above findings, the mY/mX expression ratios were negatively but not significantly correlated with synonymous site divergence (Ks) values (Supplementary Fig. S5).

Among the 34 X-specific genes, only three genes, *CpX-1*, *CpX-15*, and the pseudogene *PCpX-1*, showed expression in leaf and/or flower tissues. *CpX-1* and *PCpX-*1 showed a relatively low level of expression and *CpX-15* showed a relatively high level of expression with a transcripts per kilobase million (TPM) value calculated at 36 in female flower. *CpX-1* had nearly doubled expression in female tissues relative to male tissues, indicating that *CpX-1* is not dosage compensated. *PCpX-1* showed a higher level of expression in male flower than in female flower. *CpX-15* showed expression in male leaf and female flower tissues only, suggesting that *CpX-15* might have evolved regulatory subfunctions in different tissues.

## Discussion

Genetic degeneration is a hallmark of sex chromosome evolution^14,15,25^. The decay of genes and gene activities on the Y or W chromosome may cause expression imbalance between sexes, and could negatively affect the fitness of heterogametic sex. Dosage compensation is a process to equalize genes expression between sexes and between genes on sex chromosomes and autosomes^10,11,14,26^. To investigate whether dosage compensation operates in papaya, we conducted comparative analysis on gene expression between different sex types using RNA-Seq data generated from papaya leaf and flower tissues.

Dosage compensation has been well studied in human and mammalian systems. Compared to plant sex chromosomes, mammalian sex chromosomes are much older, evolved about 166–180 Mya^27,28^. The mammalian Y chromosome has degenerated and lost most of its original genes over evolutionary time, resulting in only a few functional genes retained^29^. Therefore, most X-linked genes are single copy in the mammalian heterogametic sex. Most dosage compensation studies in mammalian systems are based on assessment of relative expression of X-specific genes versus autosomal genes and relative expression of X-specific genes between homogametic and heterogametic sexes. However, this method cannot be easily applied into plant systems. Plant sex chromosomes are normally ‘young’ and their non-recombining regions are small^2^. In papaya, the non-recombining region of Y chromosome is 8.1 Mb and its X counterpart is 3.5 Mb^21^. A total of 50 paired X/Y genes, 34 X-specific genes and 22 Y-specific genes were annotated in the non-recombining regions of papaya sex chromosomes. Among the 34 X-specific genes, only three of them showed expression in leaf and/or flower tissues. Therefore, it is impossible to study dosage compensation by expression analysis of hemizygous X-specific genes in papaya. For this reason, we used X/Y paired genes to investigate dosage compensation in papaya. We used SNPs to distinguish X and Y alleles and used *V. monoica*, a close relative species of papaya without sex chromosomes, to normalize gene expression between sex chromosomes and autosomes.

Dosage compensation coevolved with sex chromosome degeneration. Plants with newly evolved sex chromosomes provide good opportunities to determine whether dosage compensation can evolve rapidly. Dosage compensation can be whole sex-linked region (acting on most genes in non-recombining regions) or local (acting on individual genes). In papaya, a bimodal distribution of the expression ratio of mX/fXX was observed with the main peak at 0.5 and the 2nd peak at 0.7, suggesting that dosage compensation was not established in the whole sex-linked region but might be acting on individual genes in papaya.

We further analyzed the expression of genes on different evolutionary strata. Although no dosage compensation of whole sex-linked region has been established in papaya, a gene-by-gene analysis revealed that at least seven genes were dosage regulated, including one gene (5%) in the older evolutionary stratum, four genes (21%) in the younger evolutionary stratum and two genes (13%) in the collinear region. Fewer dosage regulated genes were detected on the old evolutionary stratum, but the rate could be biased as the number of genes is too small. The Y allele of the only dosage regulated gene in inversion 1 is a pseudogene, while the Y alleles of the other five dosage regulated genes are functional genes. These genes might be dosage-sensitive genes and play critical functions. Dosage changes in these genes might cause severe deleterious effects or loss of fitness. In our study, the number of dosage regulated genes might be underestimated since dosage regulation can be achieved not only by adjustment of mRNA abundance but also by mRNA splicing, stability, or translation.

In the older evolutionary stratum, the expression level of X alleles in male was about half of that in female. And Y alleles showed reduced expression compared to their corresponding X alleles. In the younger evolutionary stratum, the expression level of X alleles in male is about half of that in female as observed in the older evolutionary stratum, but Y alleles showed relatively higher expression than their corresponding X alleles. The Y alleles diverged from X alleles as the sex chromosome evolved. They might have lost their function due to accumulated mutations but still be transcribed^21^. However, expression of a large number of malfunctional or nonfunctional Y alleles can be deleterious or costly because malfunctional transcripts may compete with the functional ones, which can lead to disturbance of normal functions. Even there is no harmful affect introduced, it is still not cost-efficient for organisms to maintain the nonfunctional transcripts. Although no significant correlation was observed between expression and *Ks* values of Y alleles, the overall expression of Y alleles in the older stratum showed a significantly higher level of reduction than the ones in the younger stratum in papaya. Due to the recombination suppression between the X and Y chromosomes, more deleterious mutations might have accumulated in Y alleles in the older evolutionary stratum than the ones in the younger evolutionary stratum^21^. Reduced expression of Y alleles in the older evolutionary stratum might be caused by accumulation of deleterious mutations in regulatory regions. Lenormand et al*.*^30^ proposed a “degeneration by regulatory evolution” (DRE) theory to explain Y chromosome degeneration. According to this theory, coding sequences carrying more deleterious mutations were selected to become associated with weak Y *cis*-elements in order to mask deleterious mutations. Reduced expression of Y alleles in the older evolutionary stratum could also be caused by transposable element (TEs)-mediated methylation spreading. TE-mediated methylation can spread beyond the TE sequence and influence the expression of nearby host genes^31^. In fact, there are more TEs accumulated in Inversion 1 than Inversion 2^21^. Immunofluorescence assay also showed a slightly higher level of methylation signal in Inversion 1 than Inversion 2^32^ Gene silencing can be another strategy to maintain transcriptional balance between sexes for the genes that could be substituted by other genes. In papaya sex-specific region, about 94.1% (32/34) of the hemizygous genes (X-specific genes) were transcriptionally silenced in leaf and/or flower tissues. The X-specific genes annotated in the papaya sex-specific region were located nearby a large highly methylated heterochromatin knob, which may change the DNA structure and suppress the expression of those genes^32^. The high percentage of silenced genes can also be the genes that have a very narrow expression span, which could escape detection due to their narrow spatial or temporal expression.

Several genes on the sex-specific region showed hyper transcription of Y alleles in papaya flower or leaf tissues. Elevated expression of Y alleles could be resulted from their specialization in male function. It can also be caused by haploid selection. Pollen experience extensive haploid selection, because they compete to access ovules to deliver the male gametes for fertilization^33^. Genes involved or expressed in haploid male gametophytes are less likely to be degenerated or lower their expression. Two genes in the inversion regions, CpXY^h^2 and CpXY^h^29, showed extremely higher expression of Y alleles. CpXY^h^2 encodes the somatic embryogenesis receptor-like kinase (SERK), and CpXY^h^29 encodes a phosphoacetylglucosamine mutase involved in DNA repair from UV damage. *AtSERK* was expressed in developing ovules and embryos in *Arabidopsis*, and it could enhance embryo competition in tissue culture^34^. Our results will lead to further investigation of haploid selection during evolution of sex chromosomes in papaya.

## Materials and methods

### Plant materials, total RNA extraction, and RNA-Seq library construction and sequencing

Papaya dioecious variety AU9 plants were grown in the field at Hawaii Agriculture Research Center Kunia Station on Oahu, Hawaii. Fully expanded leaves and flowers of three different developmental stages from male and female plants were collected for RNA extraction (Supplementary Table S1). *V. monoica* plants were grown in a greenhouse at USDA Pacific Basin Agricultural Research Center in Hilo, Hawaii. Leaf and flower tissues were collected from *V. monoica* plants for RNA extraction. Total RNA was extracted using Trizol reagent using the method as described by Lin et al.^35^. The quality and integrity of the RNA samples were determined by running on an agarose gel and using a NanoDrop 2000 (Thermo Scientific, Waltham, MA, USA). Approximately 1 µg of total RNA was used to construct RNA-Seq library using a KAPA Stranded mRNA-Seq Kit (Kapa Biosystems, Wilmington, MA, USA) following the manufacturer’s protocol. RNA-Seq libraries were quantified using a Qubit Fluorometer (Life Technologies), pooled, and sequenced on an Illumina HiSeq 2500 (Illumina, San Diego, CA, USA) at the Center for Genomics and Biotechnology, Fujian Agriculture and Forestry University. The RNA-Seq sequences are available in the SRA database of NCBI under accession number PRJNA555541.

### Gene expression analysis

Papaya male and female RNA-Seq reads were mapped on papaya male and female reference genomes^20,21,24^ using HISAT2 with default parameters^36^. The average identity of genes between papaya and *V. monoica* is about 93.1%. We therefore relaxed the stringency of mapping parameters when mapping the *V. monoica* reads on papaya genomes using HISTA2 (-p 10 --score-min L, 0.0, -0.7 --rdg 2,1 --rfg 2,1 --mp 3,1). TPM values were calculated for each library using StringTie^36^. Genes with TPM greater than 1 in at least one tissue were selected for dosage compensation analysis. TPM values of papaya genes were divided by TPM values of *V. monoica* genes individually for calibration.

### Gene expression analysis of X-specific and Y-specific alleles in the non-recombining regions of papaya sex chromosomes

To distinguish the transcript reads between X and Y alleles in male samples, we first aligned the X and Y alleles of each gene using MUSCLE^37^, and then identified SNPs between them manually. We used the reads covering the SNP sites to estimate the expression levels of X-specific and Y-specific alleles.

We mapped the RNA-Seq reads of papaya male samples onto the papaya female reference genome using HISAT2^36^. HaplotypeCaller of the GATK package was used to identify SNPs at the genome-wide level following the GATK best practice recommendations^38^. For SNPs in the non-recombining regions, the read counts of reference base and alternative base were used to estimate the expression of X-specific and Y-specific alleles, respectively. SNP loci identified in the first step were further validated whether they were homozygous in female and heterozygous in male plants. Reads matching these criteria were counted for allele-specific expression analysis. Read numbers of X and Y alleles at SNP sites within the open reading frame were summed up to calculate expression ratio between X and Y alleles. To make it comparable between libraries, the total read counts for each gene were normalized by the number of SNPs and library size using the calcNormFactors function in edgeR^39^.

## Supplementary Information

Supplementary Informations.

## Data Availability

The RNA-Seq data used in this study have been deposited and publicly available in NCBI under Accession No. PRJNA555541.

## References

[1] Hoekstra RF (2005). Evolutionary biology: why sex is good. Nature.

[2] Ming R, Bendahmane A, Renner SS (2011). Sex chromosomes in land plants. Annu. Rev. Plant Biol..

[3] Muyle A, Shearn R, Marais GA (2017). The evolution of sex chromosomes and dosage compensation in plants. Genome Biol. Evol..

[4] Disteche CM (2012). Dosage compensation of the sex chromosomes. Annu. Rev. Genet..

[5] Marais GA (2008). Evidence for degeneration of the Y chromosome in the dioecious plant *Silene latifolia*. Curr. Biol. CB.

[6] Ohno S (2013). Sex Chromosomes and Sex-Linked Genes.

[7] Mank JE (2013). Sex chromosome dosage compensation: definitely not for everyone. Trends Genetics.

[8] Walters JR, Hardcastle TJ, Jiggins CD (2015). Sex chromosome dosage compensation in Heliconius Butterflies: global yet still incomplete?. Genome Biol. Evol..

[9] Nozawa M, Ikeo K, Gojobori T (2018). Gene-by-gene or localized dosage compensation on the Neo-X chromosome in *Drosophila miranda*. Genome Biol. Evol..

[10] Jiang X, Biedler JK, Qi Y, Hall AB, Tu Z (2015). Complete dosage compensation in *Anopheles stephensi* and the evolution of sex-biased genes in mosquitoes. Genome Biol. Evol..

[11] Disteche CM (2006). Dosage compensation of the active X chromosome in mammals. Nat. Genet..

[12] Bergero R, Qiu S, Charlesworth D (2015). Gene loss from a plant sex chromosome system. Curr. Biol. CB.

[13] Muyle A (2012). Rapid de novo evolution of X chromosome dosage compensation in *Silene latifolia*, a plant with young sex chromosomes. PLoS Biol..

[14] Papadopulos AS, Chester M, Ridout K, Filatov DA (2015). Rapid Y degeneration and dosage compensation in plant sex chromosomes. Proc. Natl. Acad. Sci. U.S.A..

[15] Hough J, Hollister JD, Wang W, Barrett SC, Wright SI (2014). Genetic degeneration of old and young Y chromosomes in the flowering plant *Rumex hastatulus*. Proc. Natl. Acad. Sci. U.S.A..

[16] Krasovec M, Chester M, Ridout K, Filatov D (2018). The mutation rate and the age of the sex chromosomes in *Silene latifolia*. Curr. Biol..

[17] Chibalina MV, Filatov DA (2011). Plant Y chromosome degeneration is retarded by haploid purifying selection. Curr. Biol. CB.

[18] Krasovec M, Kazama Y, Ishii K, Abe T, Filatov D (2019). Immediate dosage compensation is triggered by the deletion of Y-linked genes in *Silene latifolia*. Curr. Biol..

[19] Muyle, A. *et al. Dioecy in Plants: An Evolutionary Dead End? Insights from a Population Genomics Study in the Silene genus*. bioRxiv 414771. 10.1101/414771 (2018).

[20] Ming R (2008). The draft genome of the transgenic tropical fruit tree papaya (*Carica papaya* Linnaeus). Nature.

[21] Wang J (2012). Sequencing papaya X and Yh chromosomes reveals molecular basis of incipient sex chromosome evolution. Proc. Natl. Acad. Sci. U.S.A..

[22] Ming R, Yu Q, Moore PH (2007). Sex determination in papaya. Semin. Cell Dev. Biol..

[23] Gschwend AR (2012). Rapid divergence and expansion of the X chromosome in papaya. Proc. Natl. Acad. Sci. U.S.A..

[24] Vanburen R (2015). Origin and domestication of papaya Y chromosome. Genome Res..

[25] Charlesworth B, Charlesworth D (2000). The degeneration of Y chromosomes. Philos. Trans. R. Soc. Lond. Ser. B Biol. Sci..

[26] Gupta V (2006). Global analysis of X-chromosome dosage compensation. J. Biol..

[27] Veyrunes F (2008). Bird-like sex chromosomes of platypus imply recent origin of mammal sex chromosomes. Genome Res..

[28] Cortez D (2014). Origins and functional evolution of Y chromosomes across mammals. Nature.

[29] Charlesworth B (1991). The evolution of sex-chromosomes. Science.

[30] Lenormand T, Fyon F, Sun E, Roze D (2020). Sex chromosome degeneration by regulatory evolution. Curr. Biol..

[31] Choi JY, Purugganan MD (2017). Evolutionary epigenomics of retrotransposon-mediated methylation spreading in rice. Mol. Biol. Evol..

[32] Zhang W, Wang X, Yu Q, Ming R, Jiang J (2008). DNA methylation and heterochromatinization in the male-specific region of the primitive Y chromosome of papaya. Genome Res..

[33] Erbar C (2003). Pollen tube transmitting tissue: place of competition of male gametophytes. Int. J. Plant Sci..

[34] Hecht V (2001). The Arabidopsis SOMATIC EMBRYOGENESIS RECEPTOR KINASE 1 gene is expressed in developing ovules and embryos and enhances embryogenic competence in culture. Plant Physiol..

[35] Lin H, Liao Z, Zhang L, Yu Q (2016). Transcriptome analysis of the male-to-hermaphrodite sex reversal induced by low temperature in papaya. Tree Genet. Genomes.

[36] Pertea M, Kim D, Pertea GM, Leek JT, Salzberg SL (2016). Transcript-level expression analysis of RNA-seq experiments with HISAT, StringTie and Ballgown. Nat. Protoc..

[37] Edgar RC (2004). MUSCLE: multiple sequence alignment with high accuracy and high throughput. Nucleic Acids Res..

[38] Van der Auwera GA (2013). From FastQ data to high confidence variant calls: the Genome Analysis Toolkit best practices pipeline. Curr. Protoc. Bioinf..

[39] Robinson MD, McCarthy DJ, Smyth GK (2009). edgeR: a Bioconductor package for differential expression analysis of digital gene expression data. Bioinformatics.

