# Supplementary material for: Transcriptional regulation of dosage compensation in Carica papaya
Source: Sci Rep. 2021 Mar 12;11:5854. doi: 10.1038/s41598-021-85480-3 (PMC7971000; doi:10.1038/s41598-021-85480-3)
Supplement: Supplementary file 1 — Supplementary Informations. [file 41598_2021_85480_MOESM1_ESM.pdf]

Transcriptional regulation of dosage compensation in *Carica papaya*

Juan Liu[1,2,3], Jennifer Han[3], Anupma Sharma[2], Ching Man Wai[3], Ray Ming[3], Qingyi Yu[2,4*]

[1]Center for Genomics and Biotechnology, Fujian Provincial Key laboratory of Haixia applied plant systems biology, Haixia Institute of Science and Technology, Fujian Agriculture and Forestry University, Fuzhou, Fujian Province, China

[2]Texas A&M AgriLife Research Center at Dallas, Texas A&M University System, Dallas, Texas, United States of America

[3]Department of Plant Biology, University of Illinois at Urbana-Champaign, Urbana, Illinois, United States of America

[4]Department of Plant Pathology & Microbiology, Texas A&M University, College Station, Texas, United States of America

* Author for correspondence: Qingyi Yu, Texas A&M AgriLife Research Center at Dallas, Texas A&M University System, Dallas, Texas, United States of America, Telephone: 972-952-9225, E-mail: qyu@ag.tamu.edu

Supplemental Table 1. Detailed information of RNAseq samples used in this study.

Sample name	Variety	Sex	Tissue	RNAseq library	Sequencing reads	Sequencing platform	Accession
F_Leaf_R1	AU9	female	fully expanded leaf	AU9_F_R1	single-end, 100nt	HiSeq2000	SAMN12321770
F_Leaf_R2	AU9	female	fully expanded leaf	AU9_F_R2	pair-end, 100nt	HiSeq2500	SAMN12321771
F_Leaf_R3	AU9	female	fully expanded leaf	AU9_F_R3	pair-end, 100nt	HiSeq2500	SAMN12321772
M_Leaf_R1	AU9	male	fully expanded leaf	AU9_M_R1	single-end, 100nt	HiSeq2000	SAMN12321773
M_Leaf_R2	AU9	male	fully expanded leaf	AU9_M_R2	pair-end, 100nt	HiSeq2500	SAMN12321774
M_Leaf_R3	AU9	male	fully expanded leaf	AU9_M_R3	pair-end, 100nt	HiSeq2500	SAMN12321775
F_Flower_R1	AU9	female	flower buds 1-6mm	AU9-F-flower1-6mm-R1	single-end, 100nt	HiSeq2000	SAMN12321776
F_Flower_R2	AU9	female	flower buds 1-6mm	AU9-F-flower1-6mm-R2	pair-end, 100nt	HiSeq2500	SAMN12321777
F_Flower_R3	AU9	female	flower buds 15-20mm	AU9-F-flower15-20mm	pair-end, 100nt	HiSeq2500	SAMN12321778
F_Flower_R4	AU9	female	flower buds 7-12mm	AU9-F-flower7-12mm-R1	single-end, 100nt	HiSeq2000	SAMN12321779
F_Flower_R5	AU9	female	flower buds 7-12mm	AU9-F-flower7-12mm-R2	pair-end, 100nt	HiSeq2500	SAMN12321780
M_Flower_R1	AU9	male	flower buds 1-6mm	AU9-M-flower1-6mm-R1	single-end, 100nt	HiSeq2000	SAMN12321781
M_Flower_R2	AU9	male	flower buds 1-6mm	AU9-M-flower1-6mm-R2	pair-end, 100nt	HiSeq2500	SAMN12321782
M_Flower_R3	AU9	male	flower buds 15-20mm	AU9-M-flower15-20mm	pair-end, 100nt	HiSeq2500	SAMN12321783
M_Flower_R4	AU9	male	flower buds 7-12mm	AU9-M-flower7-12mm-R1	single-end, 100nt	HiSeq2000	SAMN12321784
M_Flower_R5	AU9	male	flower buds 7-12mm	AU9-M-flower7-12mm-R2	pair-end, 100nt	HiSeq2500	SAMN12321785

Supplemental Table 2. The mean and median log_2 transformed expression ratios of Y alleles relative to X alleles in eight male tissues.

Order	Gene	Stratum	Mean	Median
1	CpXYh1	Inversion 1	-0.803	-0.784
2	CpXYh2	Inversion 1	3.559	3.410
3	CpXYh3	Inversion 1	-0.367	-0.353
4	PYhCpXYh5	Inversion 1	-0.814	-0.890
5	PYhCpXYh7	Inversion 1	-0.226	-0.226
6	CpXYh5	Inversion 1	-0.620	-0.684
7	CpXYh6	Inversion 1	-0.434	-0.392
8	CpXYh7	Inversion 1	-0.185	-0.111
9	CpXYh8	Inversion 1	-0.334	-0.417
10	CpXYh9	Inversion 1	-1.958	-1.971
11	PXCpXYh6	Inversion 1	-0.483	-0.600
12	PXCpXYh10	Inversion 1	-0.040	-0.150
13	PXCpXYh14	Inversion 1	-0.651	-0.705
14	CpXYh10	Inversion 1	NA	NA
15	PYhCpXYh22	Inversion 1	-0.969	-1.023
16	PXYhCpXYh1	Inversion 1	NA	NA
17	CpXYh11	Inversion 1	-1.555	-1.532
18	CpXYh12	Inversion 1	0.359	0.357
19	CpXYh13	Inversion 1	-1.233	-1.233
20	CpXYh14	Inversion 1	-1.580	-1.585
21	CpXYh15	Inversion 2	-0.005	-0.016
22	CpXYh16	Inversion 2	0.985	0.686
23	CpXYh17	Inversion 2	-0.258	-0.293
24	CpXYh18	Inversion 2	-0.131	-0.131
25	CpXYh20b	Inversion 2	2.874	2.874
26	CpXYh19a	Inversion 2	NA	NA
27	CpXYh21	Inversion 2	0.674	0.000
28	PYhCpXYh28	Inversion 2	0.113	-0.021
29	CpXYh23	Inversion 2	-2.776	-2.776
30	CpXYh24	Inversion 2	0.264	0.393
31	CpXYh25	Inversion 2	NA	NA
32	CpXYh26	Inversion 2	0.121	0.247
33	PYhCpXYh30	Inversion 2	NA	NA
34	CpXYh27	Inversion 2	0.225	0.194
35	CpXYh28	Inversion 2	0.929	1.000
36	CpXYh29	Inversion 2	3.224	3.211
37	CpXYh31	Inversion 2	0.240	-0.032
38	CpXYh32	Inversion 2	-1.544	-1.595
39	CpXYh33	Inversion 2	-0.778	-0.778
40	CpXYh34	Collinear	0.137	0.226
41	CpXYh35	Collinear	NA	NA
42	CpXYh36	Collinear	NA	NA
43	CpXYh37	Collinear	-0.233	-0.116
44	CpXYh38	Collinear	-0.411	-0.411
45	CpXYh40	Collinear	-1.222	-1.190

46	CpXYh41	Collinear	-0.038	-0.071
47	CpXYh42	Collinear	NA	NA
48	CpXYh43	Collinear	1.536	1.536
49	CpXYh44	Collinear	0.609	0.538
50	CpXYh45	Collinear	0.154	0.012
51	CpXYh47	Collinear	-0.173	-0.068
52	PXYhCpXYh4	Collinear	NA	NA
53	CpXYh49	Collinear	0.421	0.303
54	CpXYh50	Collinear	-0.096	-0.179

NA: not available. We couldn't get accurate mean or median expression ratios for some genes due to no expression or a wide dynamic range of expression in different tissues.

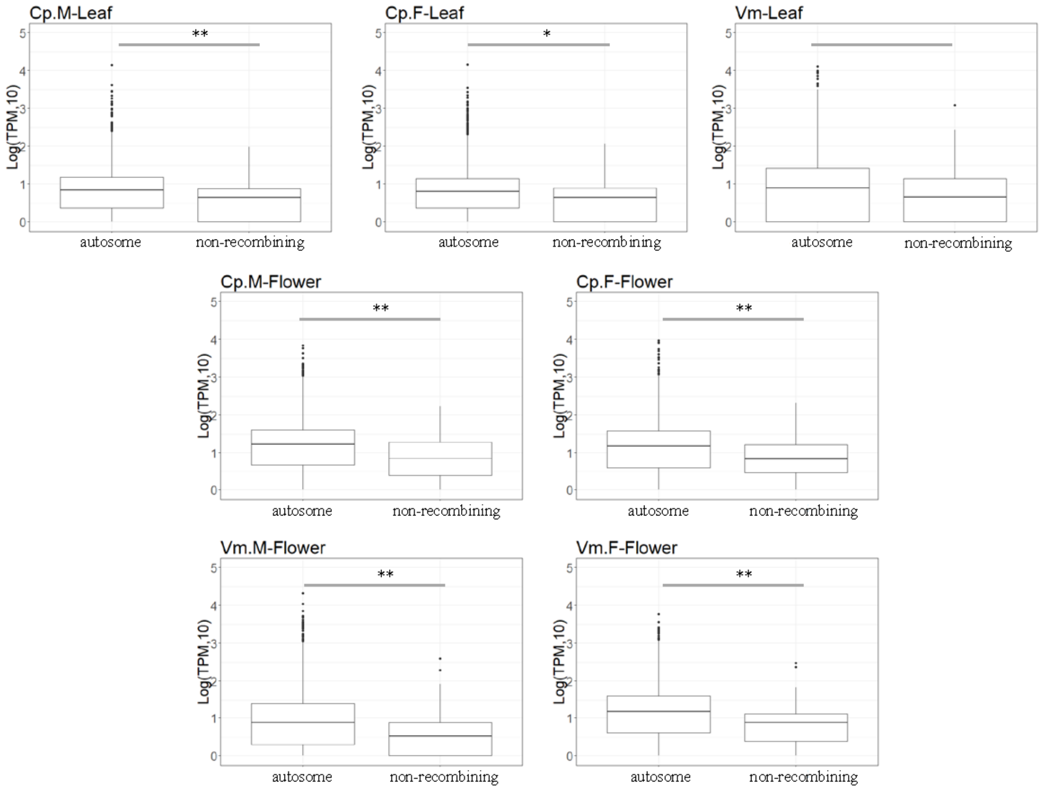

Fig. S1 Genes in sex-specific region showed a lower level of expression than autosomal genes in most samples. Cp.M-Leaf: papaya male leaf; Cp.F-Leaf: papaya female leaf; Vm-Leaf: *V. monoica* leaf; Cp.M-Flower: papaya male flower; Cp.F-Flower: papaya female flower; Vm.M-Flower: *V.monoica* male flower; Vm.F-Flower: *V.monoica* female flower. Black dots represent the outliers. Mann-Whitney' test was used for statistics analysis. ** $p < 0.01$, * $p < 0.05$, ns: no significant.

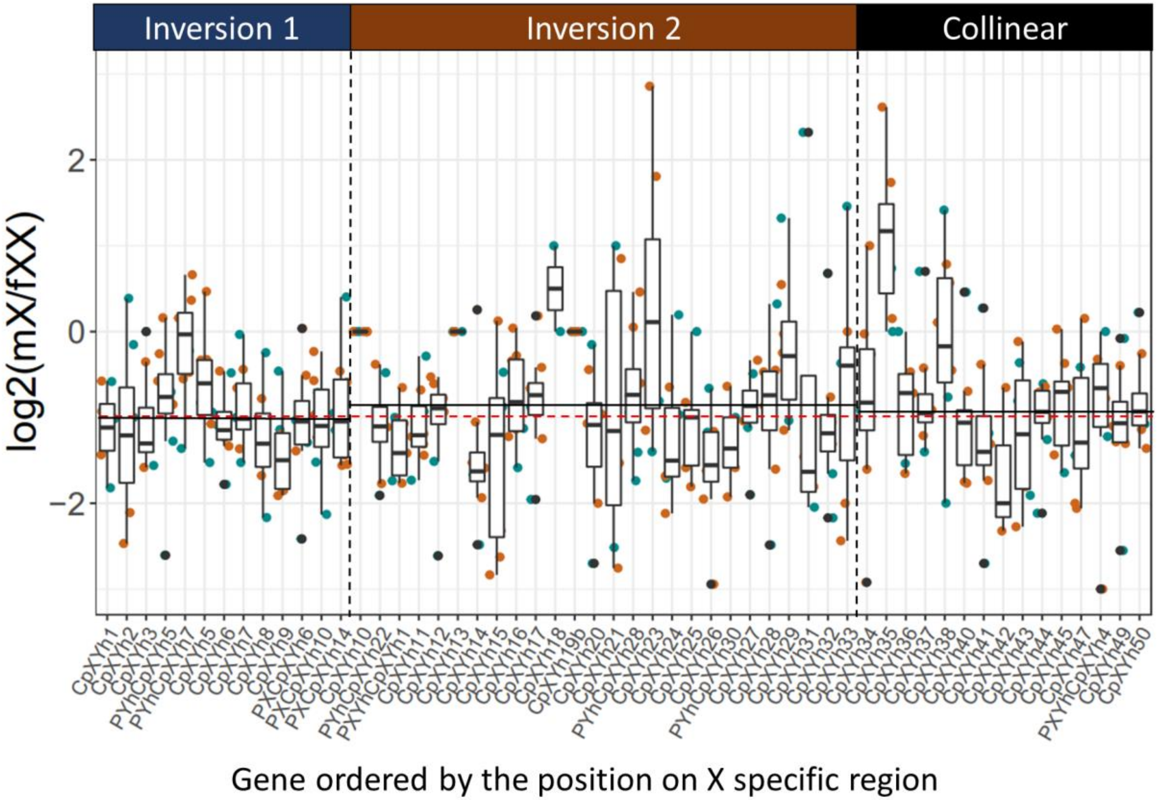

Fig. S2 Distribution of expression ratio of X alleles in male relative to female. Paired genes are displayed based on their location in X-specific region. Cyan and orange dots represent ratios in leaf and flower samples, respectively. Black dots show the outlier ratios. The red dashed line highlights the ratio of $\log_2$ (mX/fXX) at -1, at which the expression of X allele in male is half of the expression of XX in female which means no dosage compensation occurs. The black lines show the median ratios in the two evolutionary strata and the collinear region.

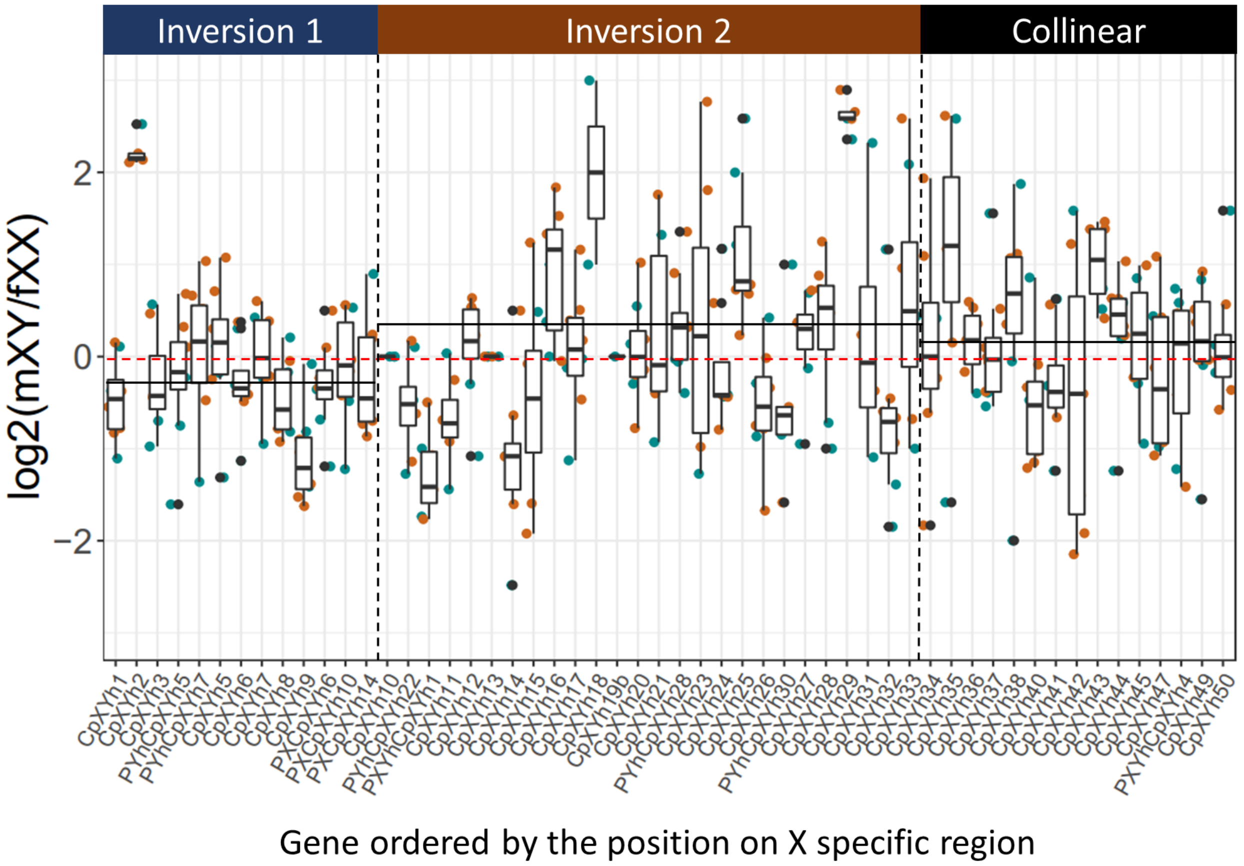

Fig. S3 Distribution of expression ratios of overall sex-specific genes between male and female (mXY/fXX). Paired genes are displayed based on their relative location in X-specific region. Cyan and orange dots represent ratios in leaf and flower samples, respectively. Black dots represent the outliner ratios. The red dashed line highlights the ratio of $\log_2$ (mXY/fXX) at 0, in which the total expression X and Y alleles in male equals to the expression of the two X alleles in female. The black lines show the median ratios in the two evolutionary strata and the collinear region.

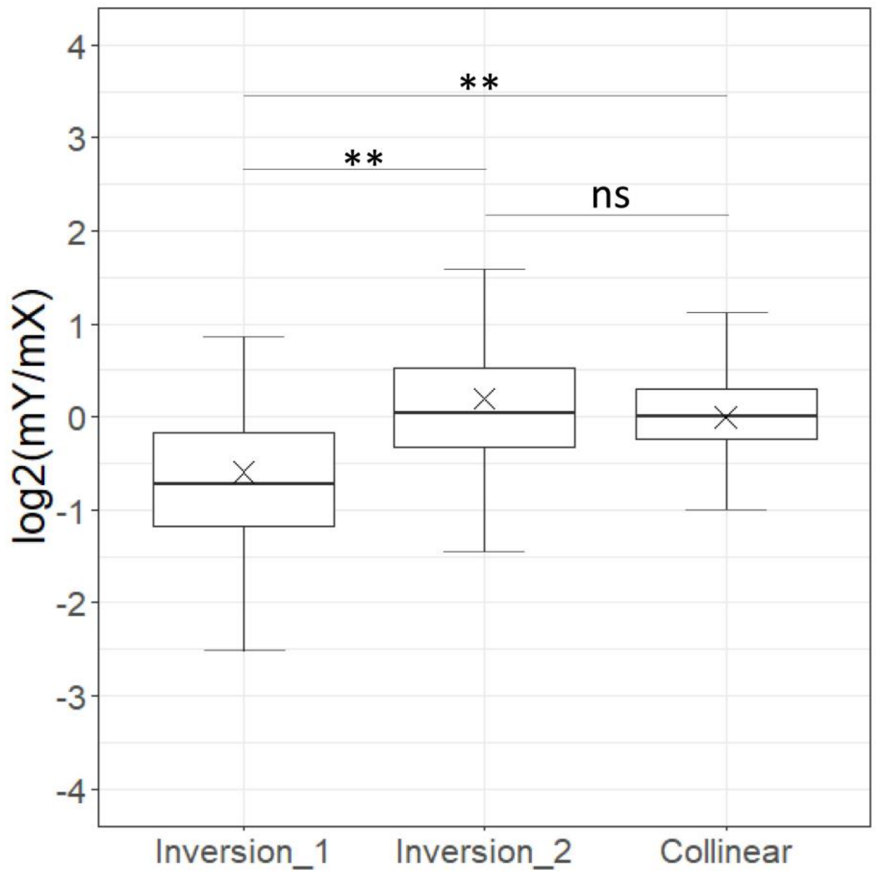

Fig. S4 Boxplot of $\log_2$ transformed expression ratio of Y/X on two evolutionary strata in papaya male samples. Mann-Whitney' test was used for statistics analysis. ** p <0.01, * p<0.05, ns: no significant.

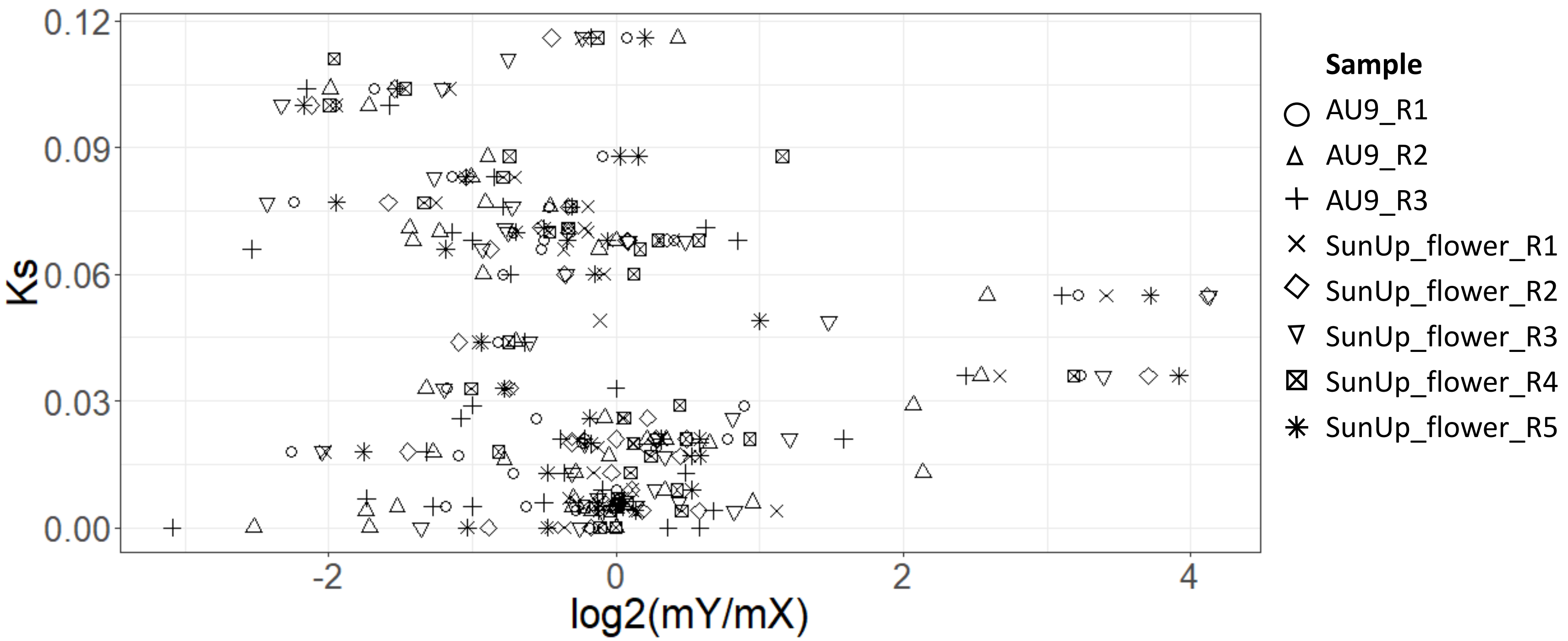

Fig. S5 Plot of $\log_2$ (mY/mX) and synonymous site divergence (Ks) of expressed genes on sex-specific regions.
